# R pyocin sensitivity of Pseudomonas aeruginosa clinical isolates from different disease types

**DOI:** 10.1099/jmm.0.002179

**Published:** 2026-06-22

**Authors:** Yanhan Deng, Meggie Wang, Chandni Chopra, Samuel A. Shelburne, William R. Miller, Natalia V. Kirienko

**Affiliations:** 1 Department of BioSciences, Rice University, Houston, TX, USA; 2 Departments of Infectious Diseases and Genomic Medicine, MD Anderson Cancer Center, Houston, TX, USA; 3 Division of Infectious Diseases, Houston Methodist Hospital, Houston, TX, USA; 4 Center for Infectious Diseases, Houston Methodist Research Institute, Houston, TX, USA; 5 Department of Medicine, Weill Cornell Medical College, New York, NY, USA

**Keywords:** *Pseudomonas aeruginosa*, pyocin sensitivity, R pyocins, serotype

## Abstract

**Introduction**. Multidrug-resistant infections by the opportunistic pathogen *Pseudomonas aeruginosa* are common in healthcare settings but are increasingly difficult to treat due to high rates of antimicrobial resistance.

**Hypothesis/Gap Statement**. Interest has developed in using R pyocins, bacteriocins produced by *P. aeruginosa*, as a species-specific treatment. We hypothesize that understanding the connections between disease type and R pyocin sensitivity will inform future treatment development.

**Aim**. To evaluate the patterns of R pyocin sensitivity across a library of 247 clinical *P. aeruginosa* isolates and compare sensitivity profiles of strains when categorizing based on disease type, O-antigen serotype and encoded R pyocin type.

**Methodology**. A combination of genomic analysis and R pyocin sensitivity assays using growth kinetics was conducted on 247 clinical isolates to evaluate genotype, phenotype and disease presentation in the context of R pyocins. Genomic analysis allowed *in silico* identification of O-antigen serotype and encoded R pyocin type, and phylogenetic comparisons revealed genetic relationships between strains.

**Results**. Comparative analysis of clinical isolates showed that certain conditions, such as chronic infection in the lungs of patients with cystic fibrosis, increased sensitivity to R pyocins. We also observed strong correlations between encoded R pyocin subtype, sensitivity and O-antigen serotype. Finally, our data showed that a large percentage of strains had shared, broad-spectrum sensitivity to R pyocins, suggesting that most clinical isolates may be treatable with R pyocins.

**Conclusion**. Our study reveals connections between disease type, O-antigen serotype and R pyocin sensitivity, which may inform future strain analysis and predict the effectiveness of R pyocin treatment.

## Data Summary

As a part of this project, 63 *P. aeruginosa* strains were sequenced. Sequences are deposited into GenBank. Sequence IDs and corresponding strain IDs are in Table S2, available in the online Supplementary Material.

## Introduction

As antimicrobial-resistant (AMR) infections continue to be a worldwide threat to human health, there is an increasing push to develop new treatments for these diseases. The Gram-negative, opportunistic pathogen *Pseudomonas aeruginosa* is ubiquitously found in soil and water [1] and employs a variety of virulence factors to colonize and thrive in the human body [2]. *P. aeruginosa* causes a variety of infections, including hospital-acquired and ventilator-associated pneumonias, urinary tract infections (UTIs) and bloodstream infections (BSIs) [3]. Importantly, *P. aeruginosa* is also a critical cause of morbidity and mortality in patients with cystic fibrosis (CF), with over 60% of adult CF patients suffering chronic *P. aeruginosa* lung infections [4,5]. However, traditional antibiotic therapies against *P. aeruginosa* are often ineffective against these infections due to intrinsic resistance factors, including the expression of efflux pumps and *β*-lactamases and the ease with which drug resistance is acquired via horizontal transfer of resistance genes [6]. These factors drive high rates of resistance to most commonly used antibiotics, including up to 30% against certain *β*-lactams like aztreonam and 40% against ciprofloxacin and levofloxacin [7]. Additionally, recent large-scale analyses of antibiotic resistance in global collections of multidrug-resistant (MDR) *P. aeruginosa* isolates revealed that even novel antimicrobial combinations, like ceftolozane/tazobactam, ceftazidime/avibactam and imipenem/relebactam, inhibited only 63%, 72% and 64% of strains, respectively [8,9].

Recently, research has turned towards developing unconventional treatments against MDR *P. aeruginosa*. Interestingly, *P. aeruginosa* employs a variety of bactericidal factors to give itself a competitive advantage, both against other bacterial species [10,13] and against other strains of *P. aeruginosa* [14]. The latter group of factors includes a group of bacteriocins known as ‘pyocins’, including the S, F and R pyocins. R pyocins, a type of tailocins, are especially of interest due to their high bactericidal activity and species specificity [15,18]. Their receptor-binding tail fibre proteins specifically bind to the core residues of LPS, with residue specificity dividing R pyocins into five subtypes, R1 through R5 [15,19]. Binding to LPS triggers a conformational change in the baseplate and sheath protein complexes, driving the core tube structure through the target cell membrane [19]. This results in ion leakage and membrane depolarization, leading to cell death. While the function of R pyocins is relatively well described, evaluations of R pyocin sensitivity have mainly been on laboratory strains such as PAO1 or PA14 [16,19], which may not fully encapsulate the way R pyocins affect broader clinical strains.

In the context of infection, R pyocins may play an important role in *P. aeruginosa* strain survival. While patients may be infected with only one strain of *P. aeruginosa*, multispecies infections are common, particularly in patients with CF [4]. Since R pyocins are species-specific, these conditions provide additional factors to consider when analysing the dynamics of R pyocin in bacterial competition. Complicating things further, different disease types result in varying infection environments, which may require unique adaptations. For example, *P. aeruginosa* strains are known to develop adaptations to persist longer than other bacterial species in chronic infections, such as in the lungs of CF patients. Long-term exposure to antibiotics and osmotic stress from the highly viscous mucus stimulates strains to quickly adapt to survive in the human host [20,23]. These environmental factors cause gene expression changes in the well-studied AlgU regulon via mutations in the *mucA* gene, resulting in a mucoid phenotype commonly found in CF lung isolates [24,25].

Therefore, when evaluating R pyocin sensitivity, disease type could shape selective pressures that result in variation in R pyocin expression. Previous evaluations of the role R pyocins play in shaping *P. aeruginosa* strain dominance focused on correlation of R pyocin type with dominant sequence types (STs) but did not consider the possible impacts of disease environments on R pyocin dynamics [17,26, 27]. Additionally, while previous research has touched on the relationship between O-antigen serotype and which of the five subtypes of R pyocin are encoded by the strain, the clinical strains that were investigated often belonged to a small subset of STs and did not consider strains that did not encode R pyocins [19,26].

In this study, we use a collection of clinically isolated *P. aeruginosa* strains to evaluate the role of disease type and serotype on R pyocin sensitivity. We found that unique disease environments, such as the CF lung, change patterns of R pyocin sensitivity and can cause loss of cognate pyocins. Furthermore, investigation of R pyocin sensitivity via genomic analysis revealed strong correlations between serotype and R pyocin sensitivity, which could inform future strain analysis and treatments.

## Methods

### Bacterial strains and growth conditions

*P. aeruginosa* was inoculated and grown in lysogeny broth (LB) (10 g l^−1^ tryptone, 5 g l^−1^ yeast extract and 5 g l^−1^ NaCl) from single colonies of strains streaked on LB agar plates (1.5% agar) at 37 °C [17]. The *P. aeruginosa* laboratory strains and clinical isolates used in this study are summarized in Table S1.

### R pyocin killing assay

To assess R pyocin-mediated inhibition in a high-throughput format, filtrate assays using growth kinetics were conducted in a 96-well format as described [17]. Notably, due to the high-throughput nature of the experiments, these strains were incubated at room temperature, as the BioTek Cytation microplate reader can hold only one 96-well plate in the temperature-controlled chamber. Growth inhibition was quantified by subtracting the OD600 of the no-R control (filtrate from the strain with an R pyocin operon deletion, PA14*Δpyocins*) from the R pyocin-treated condition at each time point. A strain was considered inhibited if ≥3 consecutive time points showed ΔOD600≤−0.15. Three biological replicates were performed.

### c.f.u. assay

For *P. aeruginosa* clinical isolates which were unable to be tested by OD-based assays due to poor growth at room temperature, c.f.u. assays were conducted to assess the bactericidal effect of R pyocins, as previously described [17]. Bacteria used in c.f.u. assays were either incubated in LB broth or synthetic cystic fibrosis sputum medium 2 (SCFM2), purchased from SynthBiome [28].

### Phylogenetic tree construction

For core-genome phylogeny construction, annotated GFF files from whole-genome DNA sequences of *P. aeruginosa* isolates were processed with nucleotide-based core gene alignment by Roary v3.12.0. A concatenated core gene alignment ALN file was generated and used to infer a maximum-likelihood phylogenetic tree with IQ-TREE v2.2.

For R pyocin tail fibre phylogeny, homologous amino acid sequences were identified using BLASTP against reference protein sequences from M0089 (R1), PA14 (R2/3/4) and 218M0087 (R5). Amino acid sequences were concatenated into a single FAA file by MAFFT v7.562 and processed by FastTree v2.2 to produce a maximum-likelihood phylogenetic tree in NWK format. The Interactive Tree of Life platform was used to visualize the tree for both analyses.

### Venn diagram generation

Venn diagrams were generated using InteractiVenn: https://www.interactivenn.net/. Statistical analysis was performed using hypergeometric analysis in R (RStudio/2025.09.2+418).

### Statistical analysis

Two-way ANOVA was conducted using GraphPad Prism. Chi-square analysis was done in GraphPad Prism and Python. Monte Carlo simulation for Fisher’s exact test was done in Python [PyCharm 2024.2.1, runtime version: 21.0.3+13-b509.11 amd64 (JCEF 122.1.9)].

### Sequencing and genomic analysis

As a part of this project, 63 *P. aeruginosa* strains were sequenced. Sequences are deposited into GenBank. Sequence IDs and corresponding strain IDs are in Table S2. Genome sequences of clinical isolates were obtained from collaborators or published studies [29]. An additional 7980 *P*. *aeruginosa* strains with draft or complete genomes from the Pseudomonas.com database were included in our analyses [30]. For identification of encoded R pyocin subtype, we used a Python script to match unique, conserved sequences of each tail fibre, as previously described [17]. *In silico* serotyping was performed using PAst 1.0, the *Pseudomonas aeruginosa* serotyping program [31].

## Results

### CF lung clinical isolates are highly susceptible to R pyocins

As mentioned above, previous research on pyocin efficiency did not consider infection type (or infection-induced bacterial adaptations) as a possible factor that may influence R pyocin sensitivity. In order to evaluate pyocin susceptibility of isolates from different disease types, we assembled a collection of 247 strains, including lung infections in patients with (*n*=63) and without (*n*=41) CF and patients with bloodstream (*n*=99), urinary tract (*n*=22) and wound (*n*=22) infections. For this panel, we performed growth kinetics with cell-free spent media, hereafter referred to as filtrate, collected from one strain representative of each of the three subtypes of R pyocin, R1, R2/3/4 (these subtypes have very high sequence similarity and are typically combined together) and R5 [16,19]. Filtrates were produced from overnight cultures and contain released R pyocins, which retain their bactericidal effect.

In order to streamline the sensitivity testing and increase assay throughput, we developed a method based on kinetic OD readings. In this assay, the growth of bacteria in the presence of filtrate with or without pyocins was compared (Fig. S1A, B). We empirically determined that an OD600 decrease of at least 0.15 units for at least three consecutive time points was indicative of pyocin sensitivity (Fig. S1C, D). This cutoff was determined based on the results of a more labour-intensive c.f.u. assay. In the c.f.u. assay, 1 h incubation of the pyocin-sensitive indicator strain *P. aeruginosa* 13s with pyocin-containing filtrates decreased bacterial count by ~10^5^ c.f.u. (Fig. S1E). Substitution of the *P. aeruginosa* 13s with the pyocin-resistant strain PA14 showed little to no difference in bacterial count after filtrate exposure. In contrast, conditions that were deemed pyocin-sensitive by the kinetic OD reading assay showed no c.f.u., corroborating spectrophotometric data (Fig. S1F).

**Fig. 1.**
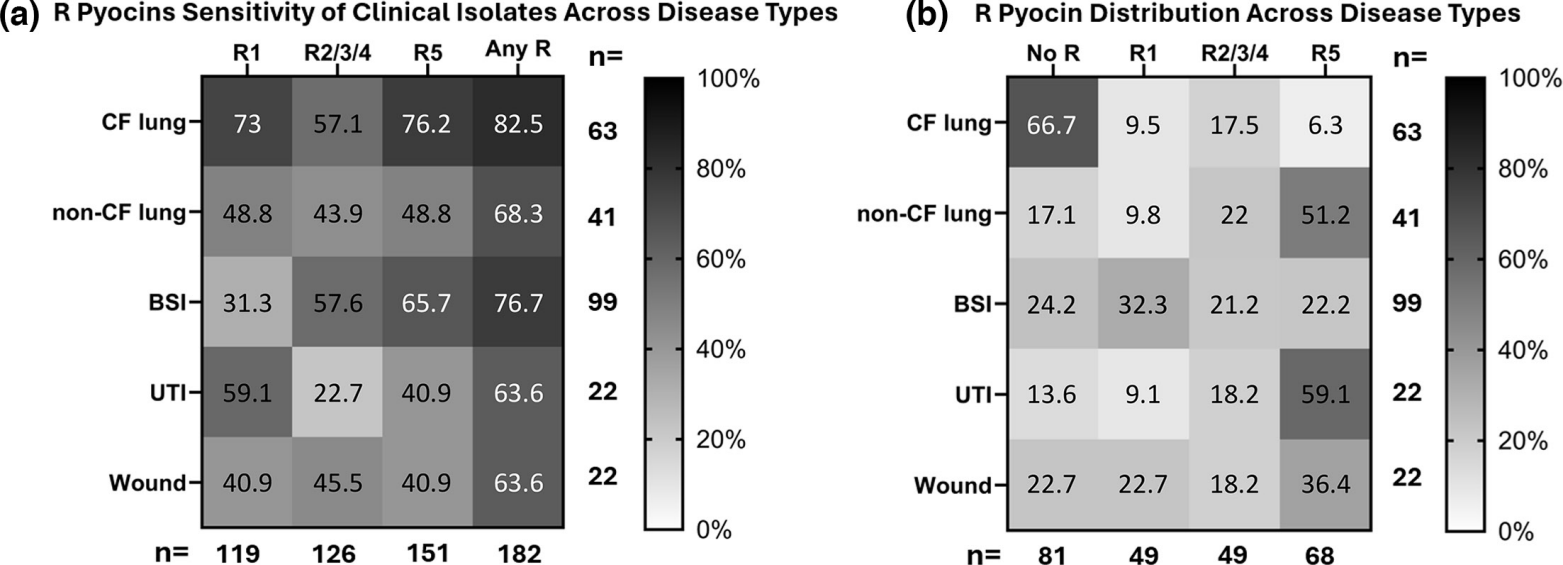
R pyocin sensitivity and distribution across clinical *P. aeruginosa* isolates categorized by infection types. (**a**) Heatmap showing the percentage of isolates from different infection types that were sensitive to R-pyocin-containing filtrates: M0089 (**R1**), PA14 (R2/3/4) and 218M0087 (**R5**). ‘Any R’ indicates sensitivity to at least one R pyocin type. Infection sources include CF lung infections, non-CF lung infections, BSIs, UTIs and wound infections. (**b**) Heatmap showing the distribution of R pyocins encoded by clinical isolates across the same infection types. ‘No R’ indicates that the isolate lacks any detected R pyocin gene cluster, and therefore does not encode any type of R pyocins. Percentages are shown by row and correspond to the proportion of isolates within each infection type. Statistical analyses can be found in Table S3.

After evaluating the R pyocin sensitivity of each strain in our clinical isolate collection, we compared sensitivity across disease types (Fig. 1a). This revealed that various *P. aeruginosa* infection types had significantly differing patterns of R pyocin sensitivity [χ^2^(12)=30.48, Cramér’s *V*=0.71, *P*=0.0024, Table S3]. Across all disease types, R1 pyocins inhibited the smallest number of strains (*n*=119), R2/3/4 (*n*=126) were intermediate, and R5 pyocins had the widest killing range (*n*=151), consistent with a previous literature report [26]. In total, 182 of 247 isolates were inhibited by at least 1 R pyocin subtype. Notably, CF lung isolates showed the broadest sensitivity to R pyocins, with sensitivity to R5 pyocins reaching 76.2%. At the other end of the spectrum was R2/3/4 pyocin sensitivity in UTI isolates (R sensitivity *P*=0.0152, disease type *P*=0.0338, Table S3). Non-CF lung and wound isolates showed very similar sensitivity for each of the three pyocin subtypes tested. Overall, these results suggest that infection environment is likely to apply different selective pressures on *P. aeruginosa*, influencing R pyocin sensitivity.

These relationships between disease type and pyocin sensitivity led us to investigate whether there was a correlation between infection type and the subtype of R pyocin encoded by the strains, as well as between autochthonous pyocin and sensitivity to pyocins of this subtype (previously, it was suggested that a strain encoding a specific subtype of R pyocin would be resistant to that subtype even if produced by a different strain) [26,32]. We used sequence analysis of the conserved tail fibre sequences to categorize the strains by R pyocin subtype (i.e. R1, R2/3/4, R5 or no R pyocin). We observed that a majority of CF lung isolates (66.7%) did not appear to encode any R pyocin, a pattern dramatically different from the strains from other disease types (Fig. 1b). R1 pyocin-encoding strains were more commonly isolated from BSIs, while R5 pyocin-encoding strains were most prevalent in non-CF lung, UTI and wound isolates. It was previously thought that across clinical isolates, R5 pyocin-encoding strains were most common [33], but our data indicate a more complex landscape, where different R pyocin strain types may dominate in different contexts. From the perspective of different disease types, the observed loss of endogenous R pyocin genes and increased pyocin sensitivity may reflect long-term, within-host adaptation during chronic infection. Overall, these results suggest that differences in the disease-specific environment may play an important role in shaping *P. aeruginosa* sensitivity to R pyocins, as well as bias in the encoded R pyocin subtype.

The chronic CF lung environment, when compared to acute infection environments, is a mucus-rich, hypoxic, nutrient-rich and often acidic habitat, which typically makes antimicrobial penetration much more difficult. Since *P. aeruginosa* strains from the sputum of CF patients were most sensitive to R pyocins, we wanted to test whether this sensitivity could be recapitulated under growth conditions that mimicked CF sputum. To mimic this chronic infection environment, we conducted pyocin sensitivity assays on a panel of isolates grown in SCFM2, designed to replicate both the turbidity and nutritional composition of CF sputum [28]. Pyocin-rich filtrates were prepared also using SCFM2 medium. The pattern of pyocin sensitivity and resistance in SCFM2 recapitulated our findings using LB (Fig. S2), suggesting that R pyocins are able to kill target bacteria in mucus-rich environments.

### R pyocin subtypes and sensitivity are correlated to serotype

As R pyocins are known to have binding specificity to residues within the LPS core, we investigated whether LPS-related factors could affect R pyocin sensitivity. One way that variations in *P. aeruginosa* LPS are reflected is through serotyping. Serotyping categorizes *P. aeruginosa* strains based on differences in the sequence and structure of the O-antigen, the outermost segment of LPS. The *P. aeruginosa* serotyping programme (PAst) allows *in silico* serotyping based on genome sequences. However, some serotypes are combined due to structural differences that are difficult to distinguish by sequence analysis alone (e.g. O5/O18/O20 group) [31].

**Fig. 2.**
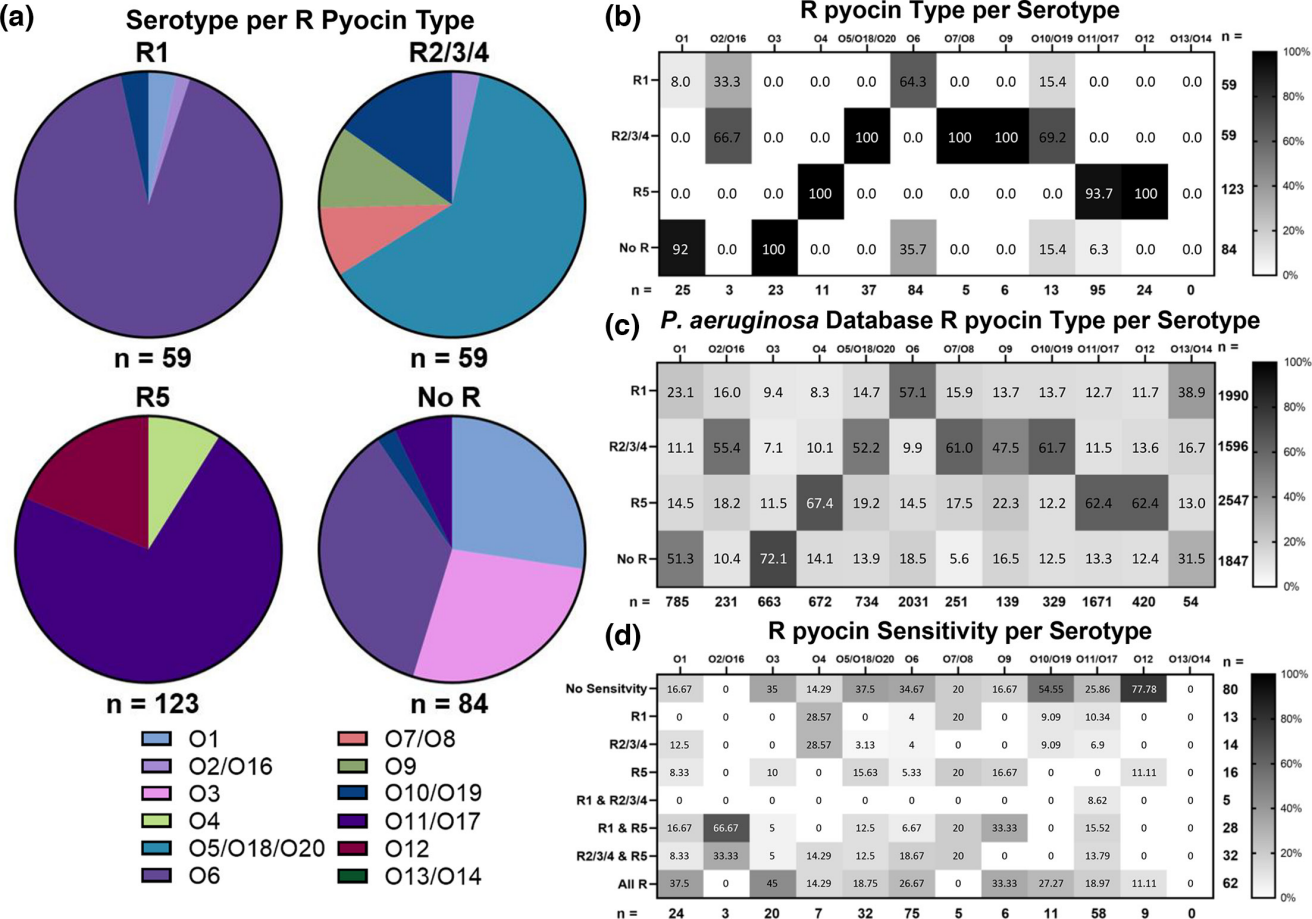
Relationship between R pyocin genotype, serotype and R pyocin sensitivity in clinical *P. aeruginosa* isolates. (**a**) Pie charts showing the distribution of *P. aeruginosa* serotypes among strains categorized by R pyocin genotype: R1 (*n*=59), R2/3/4 (*n*=59), R5 (*n*=123) and no R (*n*=84). Serotypes were assigned based on *in silico* serotyping and are colour-coded as indicated in the legend. (**b**) Heatmap showing the percentage of *P. aeruginosa* clinical isolates within each serotype (columns) that fall into each R pyocin genotype category (row). The sample size per serotype is indicated below the columns. The sample size per R pyocin type is listed vertically on the right of rows. (**c**) Heatmap showing the R pyocin type distributions by serotype using publicly available *P. aeruginosa* genome data from the *Pseudomonas* Genome Database. Each cell represents the percentage of isolates with a given R pyocin type (rows) within each serotype (columns) with the same format as **(c)**. The sample size per serotype is indicated below the columns. The sample size per R pyocin type is listed vertically on the right of rows. (**d**) Heatmap showing the percentage of isolates per serotype (column) that were sensitive to different combinations of R pyocin types (row), as determined by pyocin killing assays shown in Fig. 1(a). Categories include isolates sensitive to only one, two or all three R pyocin types. Statistical analyses can be found in Table S3.

Combining the serotyping data with our R pyocin subtyping, we investigated the different serotypes within each R pyocin subtype (Fig. 2a). For R1 pyocin-encoding strains, the largest fraction was the O6 serotype [92%, χ2(11)=138.1, Cramér’s *V*=0.46, *P*=<0.0001, Table S3], while within R2/3/4 pyocin-encoding strains, the dominant fraction was from O5/O18/O20 serotypes [63%, χ^2^(11)=277.4, Cramér’s *V*=0.66, *P*=<0.0001, Table S3]. Within R5 pyocin-encoding strains, O11/O17 serotypes were most common [72%, χ^2^(11)=278, Cramér’s *V*=0.45, *P*=<0.0001, Table S3]. Large fractions of O1, O3 and O6 serotypes were found amongst strains that do not encode any R pyocins [χ^2^(11)=101.1, Cramér’s *V*=0.33, *P*=<0.0001, Table S3]. These data suggest a relationship between serotype and encoded R pyocin subtype; this was further corroborated by analysing the different R pyocin subtypes associated with each serotype (Fig. 2b). Amongst the 325 strains (247 in our *Pseudomonas* library plus other strains for which genomic sequences were available), we observed a clear pattern, with a single R pyocin subtype being prevalent within each serotype. For example, all of our O3-serotype strains (*n*=23) encoded no R pyocins, while all of our O5/O18/O20- (*n*=37), O7/O8- (*n*=5) or O9- (*n*=6) serotype strains encoded R2/3/4 pyocins, and O4 (*n*=11) and O12 (*n*=24) strains encoded R5 pyocins (*P*=<0.0001, Table S3). Since our clinical isolate library only included a limited number of strains from each serotype (and represented geographically restricted sources), this dataset may include certain biases. To perform a more robust analysis, publicly available genome sequences for 7,980 *P*. *aeruginosa* isolates were downloaded from the *Pseudomonas* database [30], and bioinformatic analyses of the serotype and R pyocin subtype were performed (Fig. 2c). Similar patterns between R pyocin subtype and serotype were observed (albeit with greater variation). Except for the O13/O14 serotype (which was split roughly evenly between R1 and no R pyocin), each serotype had a dominant R pyocin subtype (*P*=<0.0001, Table S3). Additionally, there was substantial correlation between our dataset and the database patterns (Pearson correlation coefficient=0.9214, Table S4), suggesting that our strain library accurately represents the larger population of *P. aeruginosa* strains. Overall, these results indicate that serotype may serve as a useful proxy for R pyocin subtype.

Next, we examined the possible relationship between serotype and R pyocin sensitivity (Fig. 2d). Although there was more variability in R pyocin sensitivity, correlations between R pyocin sensitivity and serotype did appear. For example, most O10/O19 and O12 strains were resistant to all R pyocin types, while most O1 and O3 strains were sensitive to all R pyocin types. Notably, comparatively few isolates were sensitive to only R1 and R2/3/4 pyocins; only five clinical isolates of all those tested had this sensitivity profile. Sensitivity to all three pyocin subtypes was more common than sensitivity to only R1 and R5 or to R2/3/4 and R5. This challenges the current paradigm that the target range of R1 and R2/3/4 pyocins are separate subgroups of the broader range of R5 pyocins [26]. Altogether, genomic analysis of our clinical isolates reveals relationships between serotype and R pyocin subtype, and sensitivity indicates that serotyping can serve as a useful and straightforward proxy for pyocin sensitivity.

### R pyocin subtype killing spectrum

In order to evaluate the killing ability of various R pyocins within the same subtype, we selected a sub-library of 85 clinical strains for further analysis. Groups of approximately the same size (typically, five to six strains per group) were selected to represent each possible permutation of pyocin sensitivity, e.g. isolates sensitive to R1 pyocins only, isolates sensitive to R1 and R5 and isolates sensitive to R5 only (Fig. S3A). We also included 2 larger groups, 1 of 30 isolates sensitive to all pyocins and 1 group of 23 strains sensitive to none. We also noted the presence of endogenous pyocins (Fig. S3B). Importantly, these data were based on the outcome of exposing each isolate to a single pyocin from each subtype (see Fig. 1a).

Since the tail fibre sequence dictates binding specificity to LPS, we selected strains for filtrate production that would represent the largest diversity in tail fibre sequence space for each subtype. Five isolates were chosen for each R pyocin subtype, one from each major clade (Fig. S4). Each of the resulting 85 strains was treated with 16 filtrates (5 from each of the 3 R pyocin subtypes plus a control filtrate containing no R pyocins), resulting in 1,360 total conditions. As before, strains were considered sensitive if they exhibited an OD decrease of at least 0.15 units in at least three consecutive time points.

**Fig. 3.**
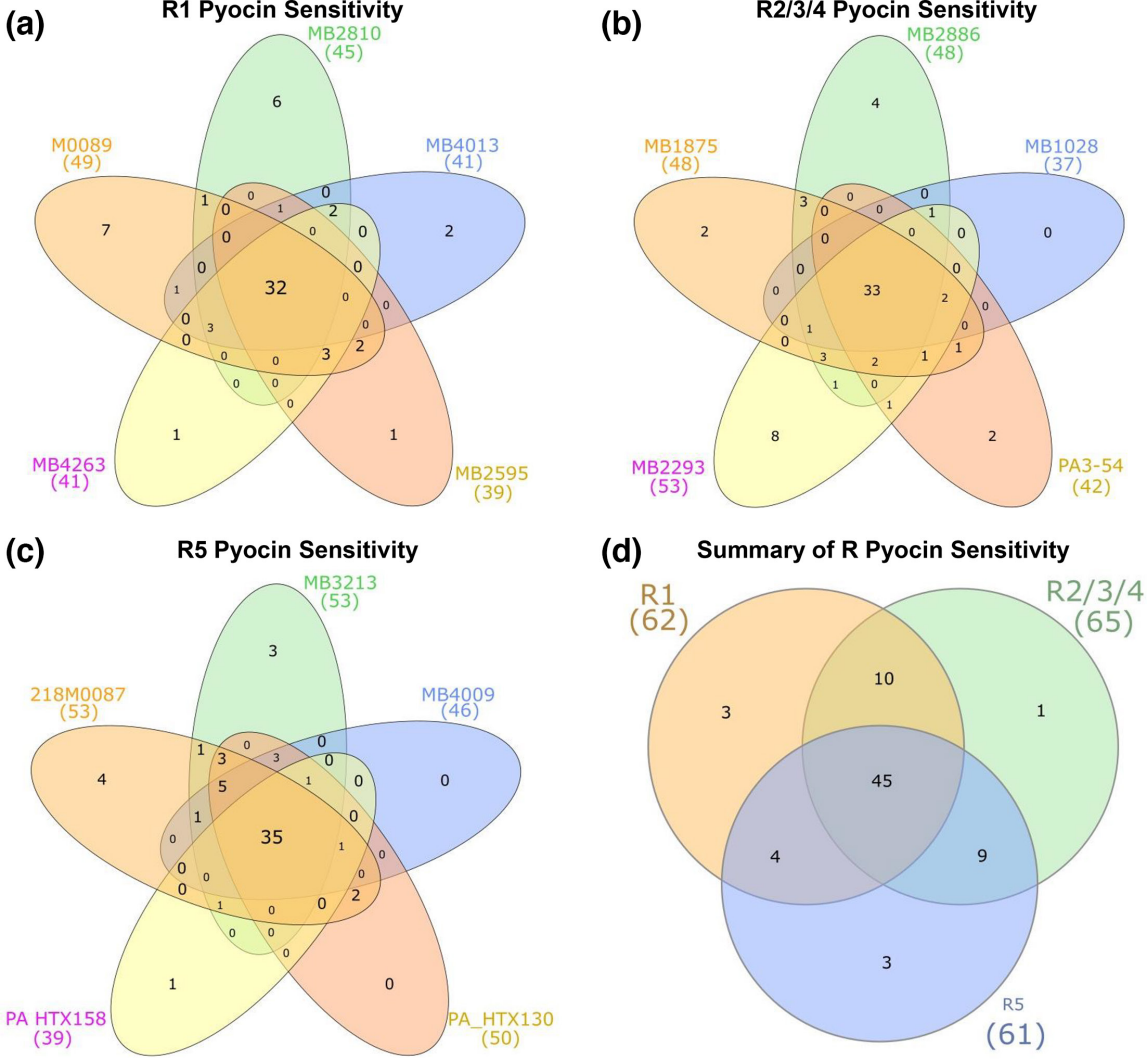
Isolate sensitivity to pyocins is shared within and between subtypes. (a–c) Venn diagrams showing the overlap in clinical isolates being inhibited by filtrates from five different R pyocin-producing clinical isolates of the same R pyocin types targeting sensitivity-based library. Library sensitivity profile is summarized in Fig. S3. Each diagram represents one R pyocin type: R1 (**a**), R2/3/4 (**b**) and R5 (**c**). The numbers in parentheses indicate the total number of strains inhibited by filtrates of each producer strain. (**d**) Venn diagram showing the overlap in sensitivity of clinical isolates to R1, R2/3/4 and R5 pyocin filtrates. For each R pyocin type, we combined the sets of isolates inhibited by any of the five corresponding filtrates, removing duplicates to avoid counting the same strain multiple times. Each R pyocin type was then treated as a single group to assess shared and unique killing activity across types. All R pyocin activities were tested against the same disease-diverse isolate collection (sensitivity library). Overlapping regions represent shared susceptible strains. All assays were done with three biological replicates. Statistical analyses can be found in Table S3.

Variations in the killing range were observed for each R pyocin subtype (Fig. 3a–c). For example, within R1 pyocins, filtrate from M0089 performed best, killing a total of 49 strains, compared to the 39 isolates killed by the least effective filtrate, from MB2595 (Fig. 3a). Of the isolates tested, 32 were killed by each of the R1 pyocin-containing filtrates. Similar patterns were observed for R2/3/4 pyocin-containing filtrates, with MB2293 killing 53 strains, and MB1028 killing only 37, but with 33 isolates killed by all R2/3/4 filtrates (Fig. 3b). Finally, within the R5 pyocin filtrates, 218M0087 inhibited 53 strains, while PA_HTX158 inhibited 39. Overall, 35 clinical isolates were sensitive to all tested R5 filtrates (Fig. 3c). Within each subtype of R pyocin, while there was some variation between the killing spectrum of the different filtrates, the largest category was strains targeted by all three filtrates. This suggests that the killing range across differing tail fibres within the subtype tends to be the same. Slight variation in killing spectra within subtypes could be a consequence of different R pyocin tail fibre sequences that, in turn, change binding affinity to the targeted residues, or differing concentrations of pyocins produced by these strains. Next, we examined the overlap in killing ranges between clades. For simplicity, we considered a strain inhibited by any R1 pyocin tested to be sensitive to R1 pyocins in general. (The same simplification was made for R2/3/4 and R5 pyocins.) Based on this criterion, 45 strains were inhibited by all 3 subtypes (Fig. 3d). When combined, the killing spectrum of the five R2/3/4 pyocins (*n*=65) was very similar to that of the R1 (*n*=62) or R5 pyocin (*n*=61) filtrates (*P*=0.3523, Table S3).

### R pyocin sensitivity across phylogeny-based library

**Fig. 4.**
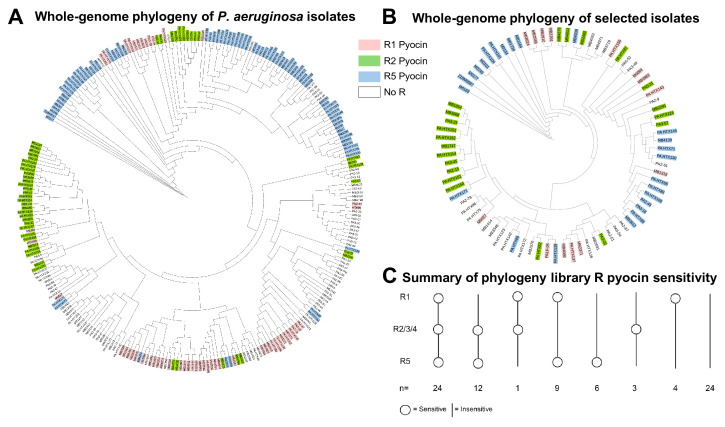
Whole-genome phylogeny of clinical *P. aeruginosa* isolates. (**a**) Whole-genome phylogeny of 265 clinical *P. aeruginosa* isolates. Strains are colour-coded by R pyocin subtypes: R1 (pink), R2/3/4 (green), R5 (blue) and no R pyocin (white). (**b**) Subset of 83 isolates selected for phylogeny library, chosen solely to capture maximal phylogenetic diversity across the tree in (**a**). R pyocin types of clinical isolates and sensitivity profiles of the library are not factors that influence selection of clinical isolates. (**c**) Summary of phylogeny library isolates by their sensitivity profiles to R1, R2/3/4 and R5 pyocins. Each vertical column represents a unique combination of sensitivities, with circles being sensitive and lines being resistant. The number of isolates per category is shown below. The phylogeny-based library was curated to reduce phylogenetic redundancy and represent the widest phylogenetic diversity, enabling efficacious testing of R pyocin specificity across diverse *P. aeruginosa* lineages.

To develop this first sub-library of strains, the only criterion used was pyocin sensitivity, with strains chosen to represent each potential sensitivity (Figs 3 and S3). Since our results that all pyocin clades largely converge on the same target strains were unexpected, we aimed to use an orthogonal library to validate these results. A phylogenetic tree of 247 clinical *P. aeruginosa* isolates was generated based on whole genomes (Fig. 4a; actual phylogenetic distances are shown in the tree in Fig. S5). This tree included the 247 strains previously investigated and added 18 BSI isolates from Oregon Health and Science University [34]. Strains in the tree were colour-coded based on which pyocin subtype each strain was predicted to encode. Using this map, we selected 83 strains to accurately represent the overall genome phylogeny (phylogenetic library, Fig. 4b; actual phylogenetic distances are shown in the tree in Fig. S6). Although these strains were chosen on the basis of phylogeny rather than R pyocin sensitivity, categories of R pyocin sensitivity across the 83 strains were comparable to the sensitivity-based sub-library (Fig. 4c, Table S3).

**Fig. 5.**
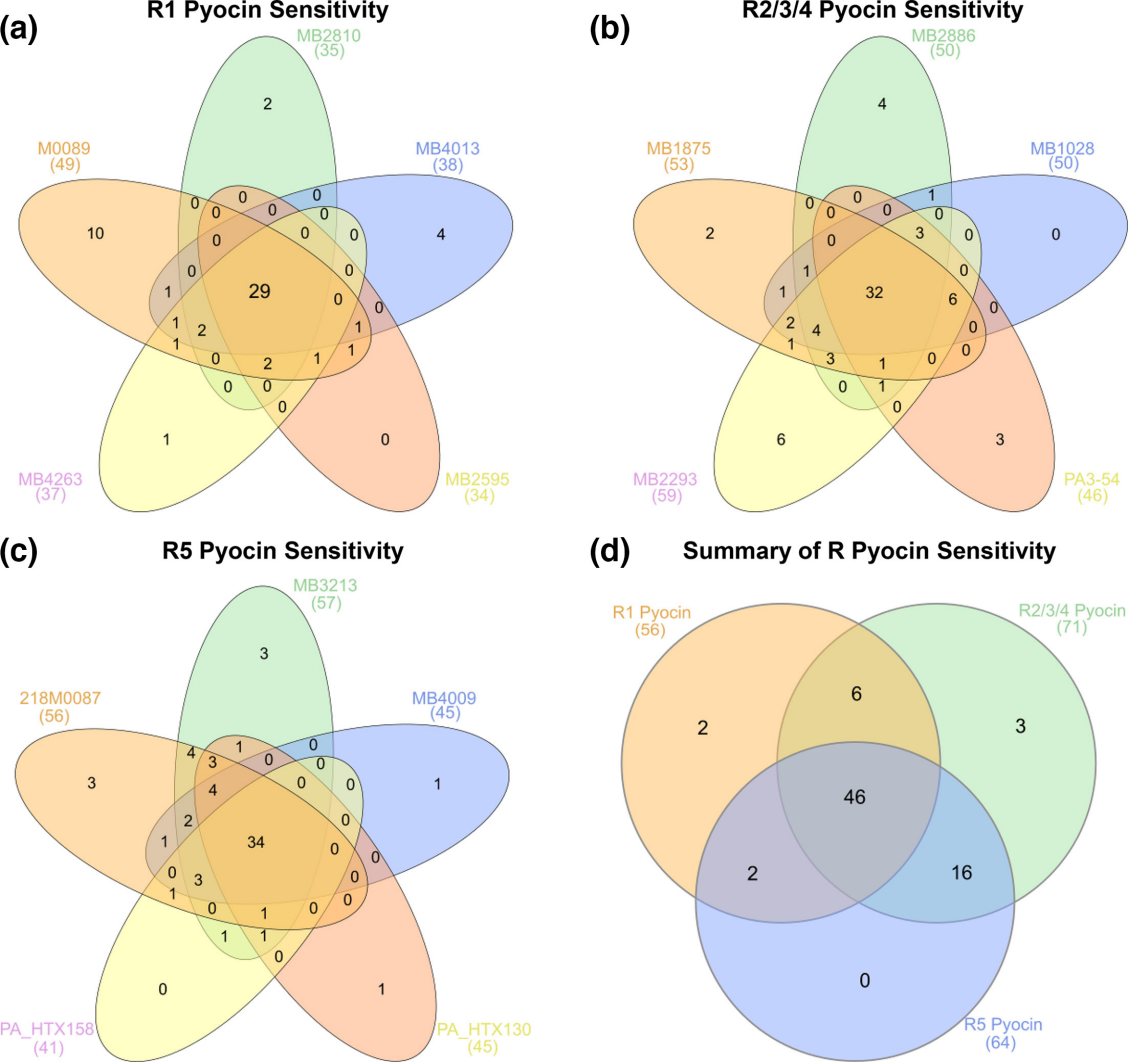
Phylogenetically selected isolates corroborate shared pyocin sensitivity within and between subtypes. (a–c) Venn diagrams showing the overlap in clinical isolates being inhibited by filtrates from five different R pyocin-producing clinical isolates of the same R pyocin types targeting the phylogeny library. (**d**) Venn diagram showing the overlap in sensitivity of clinical isolates to R1, R2/3/4 and R5 pyocin filtrates. All R pyocin activities were tested against the same disease-diverse isolate collection (phylogeny library). Overlapping regions represent shared susceptible strains. All assays were done with three biological replicates. Statistical analyses can be found in Table S3.

The phylogenetic sub-library was exposed to the same 16 filtrates (5 from each of 3 subtypes and a no R pyocin filtrate control), resulting in a total of 1,328 conditions (Fig. 5a–c). As before, the largest category within each R pyocin subtype was comprised of strains targeted by all five filtrates. In addition, when killing ranges between clades were analysed, 46 out of 83 strains (55%) were targeted by at least 1 pyocin from each clade, and 75 out of 83 strains (90%) were targeted cumulatively (Fig. 5d). Notably, a comparable number of strains were inhibited by all three filtrate groups between the two sub-libraries (45/85 vs. 46/83), suggesting that both methods adequately predict R pyocin sensitivity for clinical isolates. As before, we observed that R2/3/4 pyocins cumulatively killed the largest number of strains (*n*=71), with R5 (*n*=64) killing smaller numbers, and R1 (*n*=56) killing the least (combined *P*=0.0136, Table S3). Altogether, the results from our two clinical isolate sub-libraries show that R pyocins within or between clades tend to target a shared pool of sensitive strains, with less variability in the killing spectrum of R pyocin subtypes than previously reported [26,35].

## Discussion

Given the continued emergence of antimicrobial resistance in the pathogen *P. aeruginosa*, there is a need for novel therapeutic strategies with subsequent increasing interest in R pyocins. However, knowledge of both R pyocin activity and sensitivity across a range of clinical *P. aeruginosa* strains is limited. Using a collection of 247 *P*. *aeruginosa* clinical isolates, we performed genomic analysis and evaluated R pyocin sensitivity to determine whether connections could be drawn between disease type, serotype and phylogenetic sequence differences. By categorizing strains based on disease type, we were able to identify trends in pyocin sensitivity unique to CF lung isolates. Unlike other disease environments, the CF lung poses a unique challenge for strain survival. Extensive use of antibiotics to combat chronic infections, co-infection and competition from other present bacterial species, chronic inflammation and immune system surveillance and the osmotic pressures of the thick mucus all require *P. aeruginosa* to make adaptations to successfully colonize and infect this niche. While R pyocins provide a clear competitive advantage in environments with multiple *P. aeruginosa* strains, the energetic cost of producing a large protein complex such as the R pyocin becomes disadvantageous if many of the other commonly present in CF lung bacteria, like *Staphylococcus aureus* and *Burkholderia* spp., are insensitive to pyocins. We hypothesize that these selection pressures may push strains to lose R pyocin encoding genes. This is consistent with our observations that isolates from CF isolates are more likely to lack R pyocin genes than other disease types investigated.

Perhaps unsurprisingly, CF isolates also showed increased sensitivity to R pyocins. Strains that produce R pyocins must also possess some method of preventing R-pyocin-mediated autolysis. Our analysis indicates that the method used typically protects the strain from other R pyocins from the same clade. We think it is likely that the same pressures that contribute to the loss of R pyocins also cause the loss of the protective measure. This suggests that a more thorough investigation into the adaptations that occur in CF lung isolates may lead to interesting discoveries about the causes of R pyocin sensitivity and protection. Future studies should also investigate the correlations between well-described LPS changes in CF lung and LPS changes in R pyocin-sensitive CF isolates.

Next, we investigated the correlation between serotype and R pyocin sensitivity. Categorizing *P. aeruginosa* by O-antigen sequence and structure is routine, and previous studies have drawn connections between which R pyocin subtype is encoded by each strain and its respective O-antigen serotype, but few studies have investigated how serotype could affect sensitivity to R pyocins [19,26]. Since R pyocins are known to target the core residues of LPS [19], changes to the structure of the O-antigen could affect the accessibility of said residues. Our study revealed clear patterns between serotype and which R pyocin subtype each strain encodes, as well as serotype and R pyocin sensitivity. This connection may allow rapid serotype-based prediction of strain sensitivity to pyocins, thereby implicating which R pyocin treatment would be most effective on a strain-to-strain basis in both research and clinical settings [36].

Finally, we evaluated the R pyocin sensitivity for a panel of clinical isolates using filtrates from other clinical isolates. We saw no clear difference in killing range amongst phylogenetically different strains, with most strains being sensitive to any of the five strains within each subtype. Additionally, when comparing the R1, R2/3/4 and R5 clades to each other, there was a large overlap of strains that were inhibited by each clade. These results were inconsistent with previous reports that R5 pyocins had a wider killing range than other R pyocin subtypes [26,35], likely due to having a narrow focus on strains, typically only evaluating CF isolates, for which we do observe increased R5 pyocin sensitivity for. With a more holistic collection of clinical strains, we show that the efficacy of each R pyocin subtype we evaluated was equivalent against clinical isolates, that the killing spectrum for each strain within R pyocin subtypes was similar and that a smaller fraction of strains exhibit partial R pyocin resistance. Importantly, this means that the majority of clinical strains can be targeted by most R pyocins from any clade. Interestingly, pyocin sensitivity does not seem to be correlated with the antimicrobial susceptibility of clinical isolates used in this study (Table S5) [34,37]. While CF isolates are often MDR, we did not observe any relationship between pyocin sensitivity and drug resistance. However, since the number of isolates for each disease type was somewhat limited, some of the relationships could be missed due to the group size limitation or the presence of the confounding variables.

As the need for new, effective treatments against AMR infections increases, the bactericidal effects of R pyocins become an increasingly tempting option. Although evidence *in vivo* remains limited, several researchers have begun to evaluate the use of R pyocins in murine lung infection models [35,38]. Promisingly, intravenous or intraperitoneal injection with R pyocins has shown considerable effectiveness without significant overt inflammatory response [39].

Another critical area of exploration is the evaluation of the propensity of *P. aeruginosa* to develop novel resistance to formerly effective R pyocins during short- and long-term clinical use. For example, numerous LPS-related mutations arise during chronic infections [20]; the potential for these mutations (or others) to reduce pyocin effectiveness is a critical question. Understanding the factors that influence R pyocin sensitivity is a critical step in making it possible to rapidly predict effective, tailored treatment for *P. aeruginosa* infection.

## Supplementary material

10.1099/jmm.0.002179 Supplementary Material 1.

10.1099/jmm.0.002179 Supplementary Material 2.

## Abbreviations

AMR: antimicrobial-resistant
BSIs: bloodstream infections
CF: cystic fibrosis
MDR: multidrug-resistant
SCFM2: synthetic cystic fibrosis sputum medium 2
STs: sequence types
UTI: urinary tract infection
