# Supplementary material for: R pyocin sensitivity of Pseudomonas aeruginosa clinical isolates from different disease types
Source: J Med Microbiol. 2026 Jun 22;75(6):002179. doi: 10.1099/jmm.0.002179 (PMC13286288; doi:10.1099/jmm.0.002179)
Supplement: Supplementary Material 1. [file jmm-75-02179-s001.pdf]

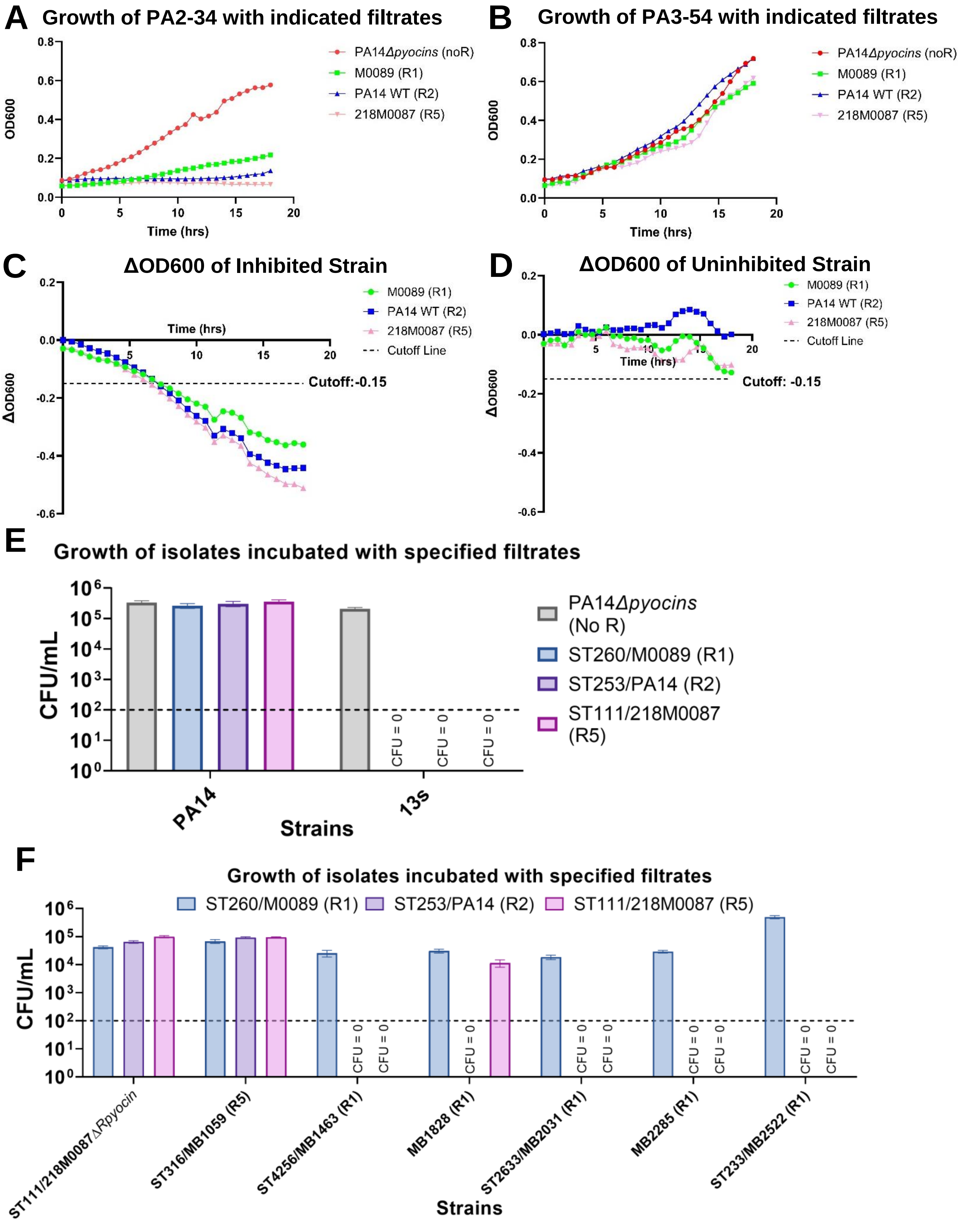

Figure S1. Growth inhibition of clinical isolates under R pyocin-containing filtrates. (A) Growth kinetics of PA2-34 exposed to filtrates from strains producing different R pyocin subtypes (M0089 R1, PA14 R2/3/4, 218M0087 R5) compared to the no-R pyocin control (PA14Δ*pyocins*). PA2-34 shows strong inhibition by all 3 types of R pyocins. **(B)** Growth kinetics of PA3-54 under the same conditions shows no appreciable inhibition, with all curves overlapping the no-R control. **(C)** Growth inhibition of PA2-34 quantified as ΔOD600 (OD600 of no-R – R pyocin condition). All three R pyocin types tested (R1, R2/3/4, R5) yield ΔOD600 curves below the cutoff threshold (–0.15) for more than 3 consecutive time points, confirming PA2-34 as a "hit." **(D)** Growth inhibition of PA3-54 measured in the same manner shows no significant inhibitions, with values remaining near baseline above the cutoff threshold, indicating resistance to all 3 types of R pyocins. **(E)** Colony-forming unit (CFU) assay for R pyocin-sensitive indicator strain 13s. **(F)** CFU assay for strains that do not grow well under room temperature conditions. Results confirm R pyocin activity against susceptible strains, with resistant strains maintaining high CFU counts.

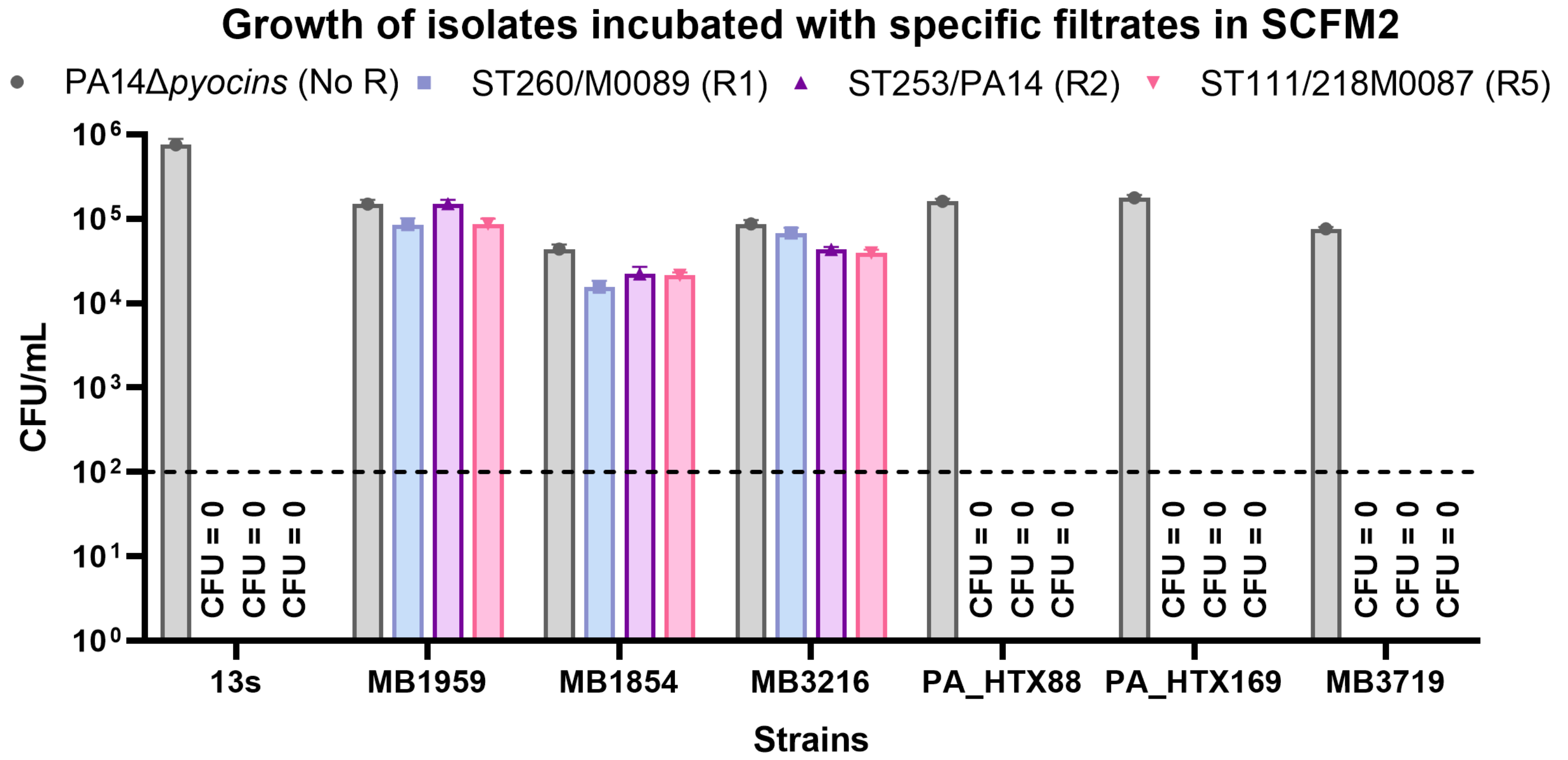

Figure S2. Growth of isolates incubated with specific filtrates in SCFM2. (A) Colony-forming unit (CFU) assay of strains incubated in synthetic cystic fibrosis medium 2 (SCFM2). Results confirm R pyocin activity against sensitive strains in turbid media, similar to the environment found in CF sputum. MB1959, MB1854, and MB3216 are also observed to be resistant in both LB and SCFM2, and MB1147, MB1540, and MB3719 are observed to be sensitive in both LB and SCFM2. Detection limit of CFU assay (horizontal dashed line) is 100 colonies/mL per individual technical replicate. Error bars represent SEM.

A **Summary of R pyocin Sensitivity of Sensitivity-based Library Isolates**

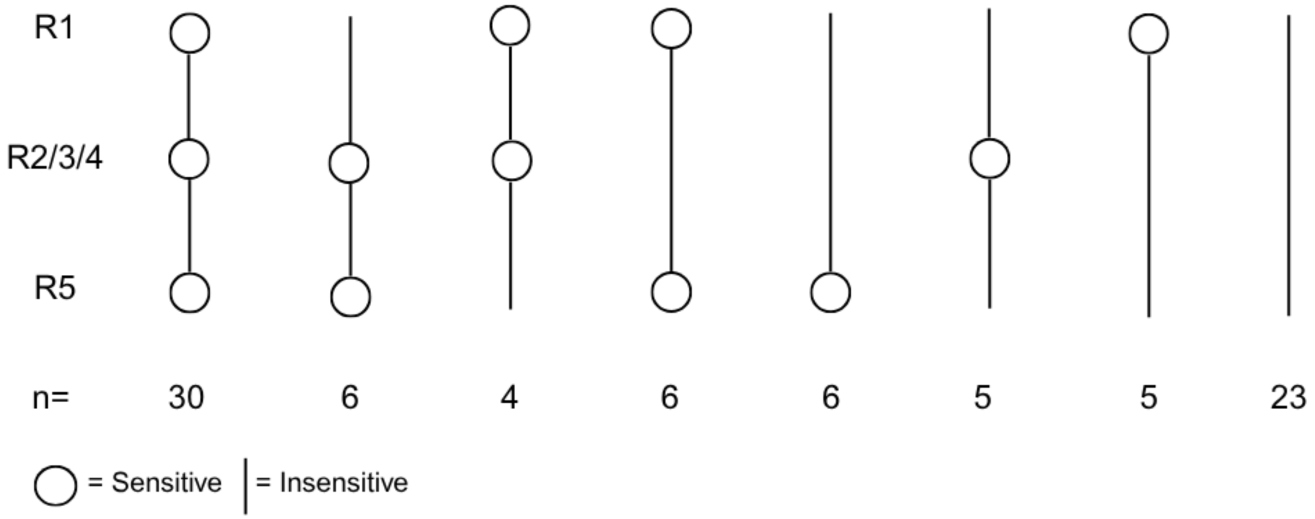

B **Categories informing strain selection of Sensitivity-based Library**

# of strains	Description
1	13s
1	PA14
1	PA14ΔwaaL
1	PA14ΔpyocinΔwaaL
1	M0087
1	M0087ΔwaaL
15	encode no R, sensitive to all R pyocins
5	encode R1, sensitive to all R pyocins
4	encode R2/3/4, sensitive to all R pyocins
6	encode R5, sensitive to all R pyocins
5	encode no R, resistant to all R pyocins
6	encode R1, resistant to all R pyocins
6	encode R2/3/4, resistant to all R pyocins
6	encode R5, resistant to all R pyocins
6	sensitive to R1 & R5 only (can encode any or no R pyocin)
6	sensitive to R2 & R5 only (can encode any or no R pyocin)
4	sensitive to R1 & R2 only (can encode any or no R pyocin)
5	sensitive to R1 only (can encode any or no R pyocin)
5	sensitive to R2 only (can encode any or no R pyocin)
6	sensitive to R5 only (can encode any or no R pyocin)

Figure S3. Sensitivity of selected strains to R pyocin subtypes. (A) Sensitivity-based Library composition. Each vertical column represents a unique combination of sensitivities, with circles being sensitive and lines being resistant. **(B)** The number of isolates per category.

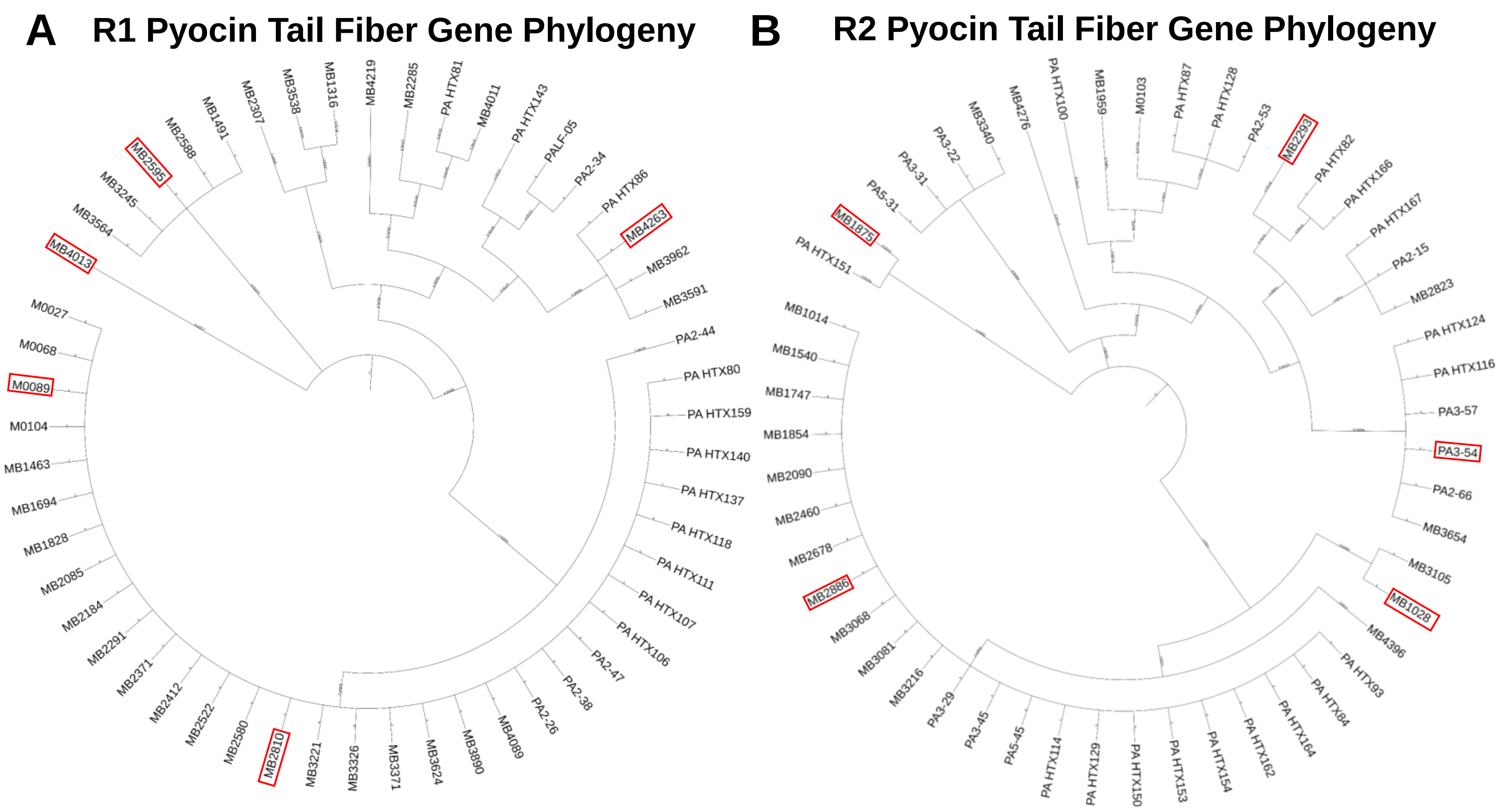

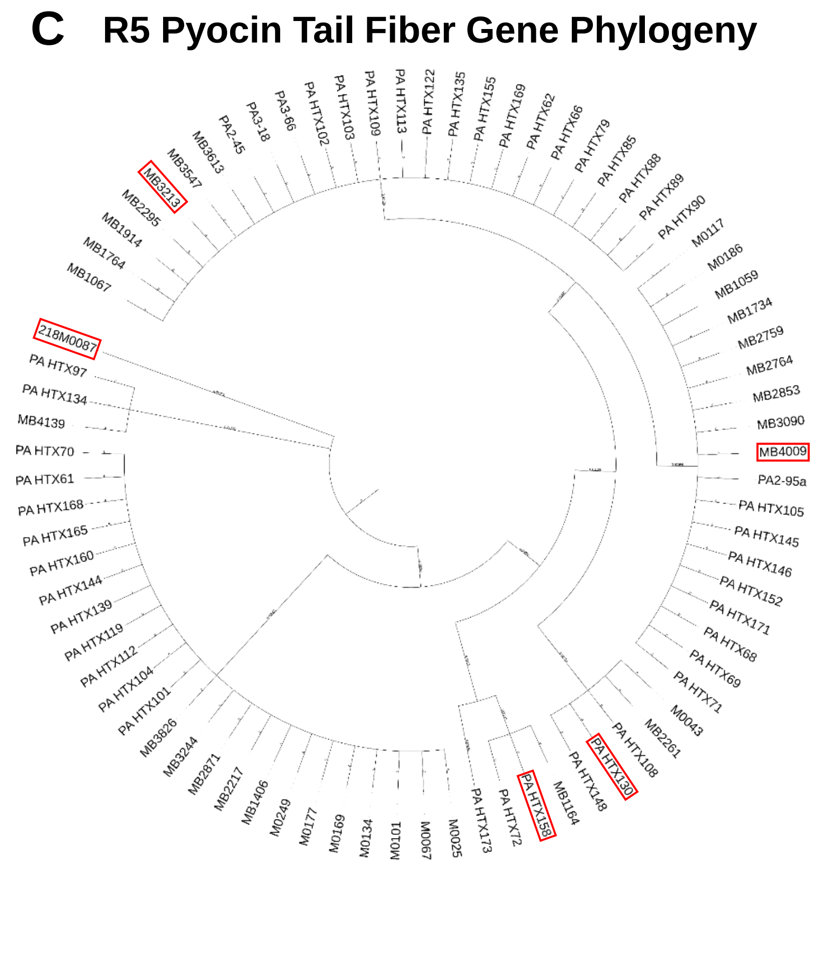

Figure S4. Phylogeny of R-pyocin tail fiber genes in *P. aeruginosa*. (A) Phylogenetic tree based on tail fiber genes of R1-pyocin-producing strains. **(B)** Phylogenetic tree based on tail fiber genes of R2/3/4-pyocin-producing strains. **(C)** Phylogenetic tree based on tail fiber genes of R5-pyocin-producing strains. The trees show relationships among strains that produce the same R-pyocin subtype. The five strains highlighted in red were intentionally chosen as filtrate producers to maximize phylogenetic spread. These filtrates were used to screen clinical-isolate library plates to (i) test how phylogenetically distinct producers within a given subtype differ in inhibitory activity and (ii) compare inhibition across subtypes (R1 vs R2/3/4 vs R5) against the same isolate panel.

A **Whole-genome phylogeny of *P. aeruginosa* clinical isolates**

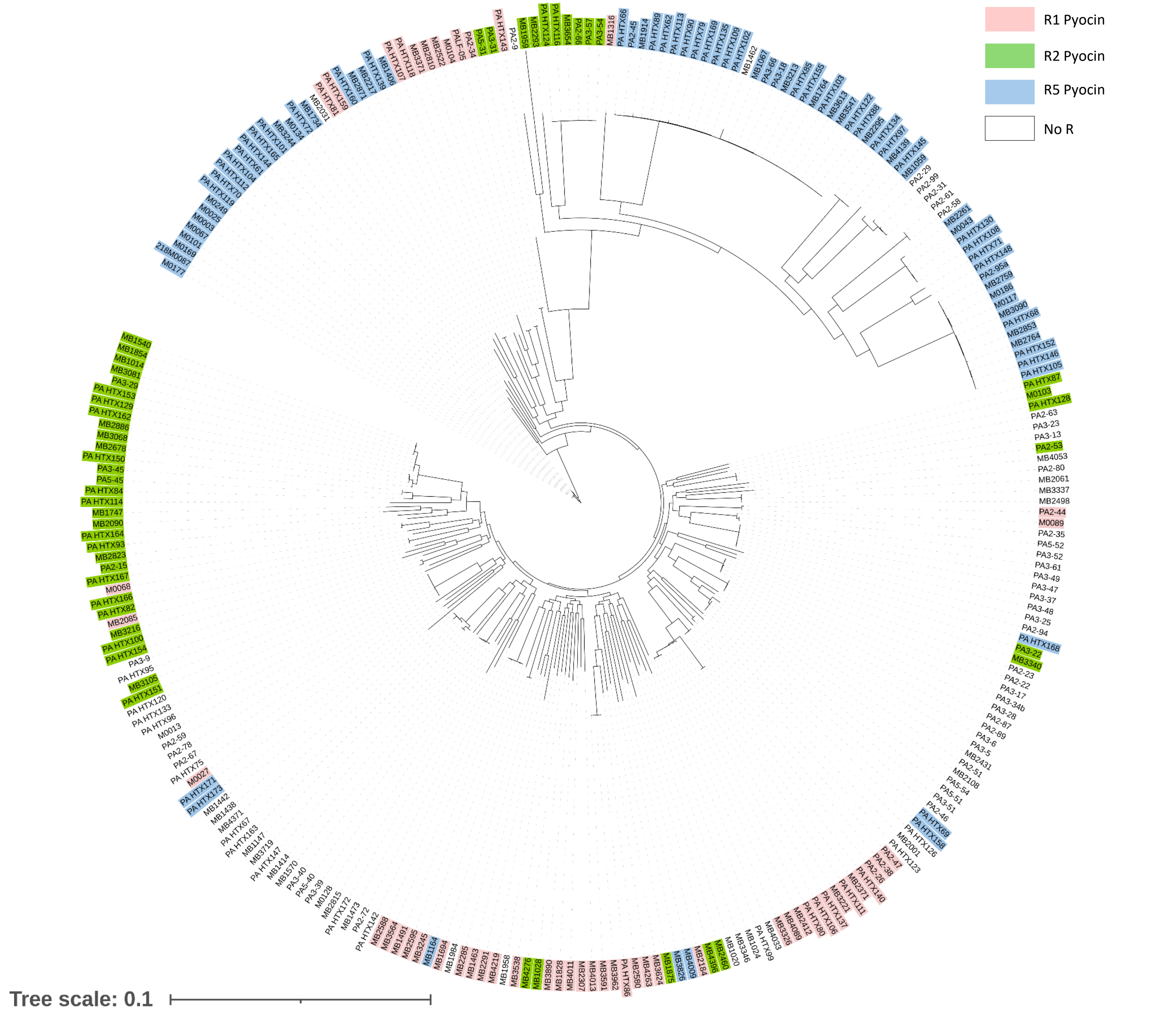

Figure S5. Branch length–scaled whole-genome phylogeny of 265 clinical *P. aeruginosa* isolates. Circular maximum-likelihood whole-genome tree generated from the core-genome SNP alignment (see Methods). Branch lengths are proportional to substitutions per site (scale bar = 0.1). Tip labels are highlighted in different colors by R pyocin subtype encoded by each isolate—R1 (pink), R2/3/4 (green), R5 (blue), and no R (white). This supplemental figure displays the true branch-length differences of 265 clinical *P. aeruginosa* isolates; the main-text figure is a topology-only cladogram (branch lengths not to scale) for readability.

A **Whole-genome phylogeny of selected isolates**

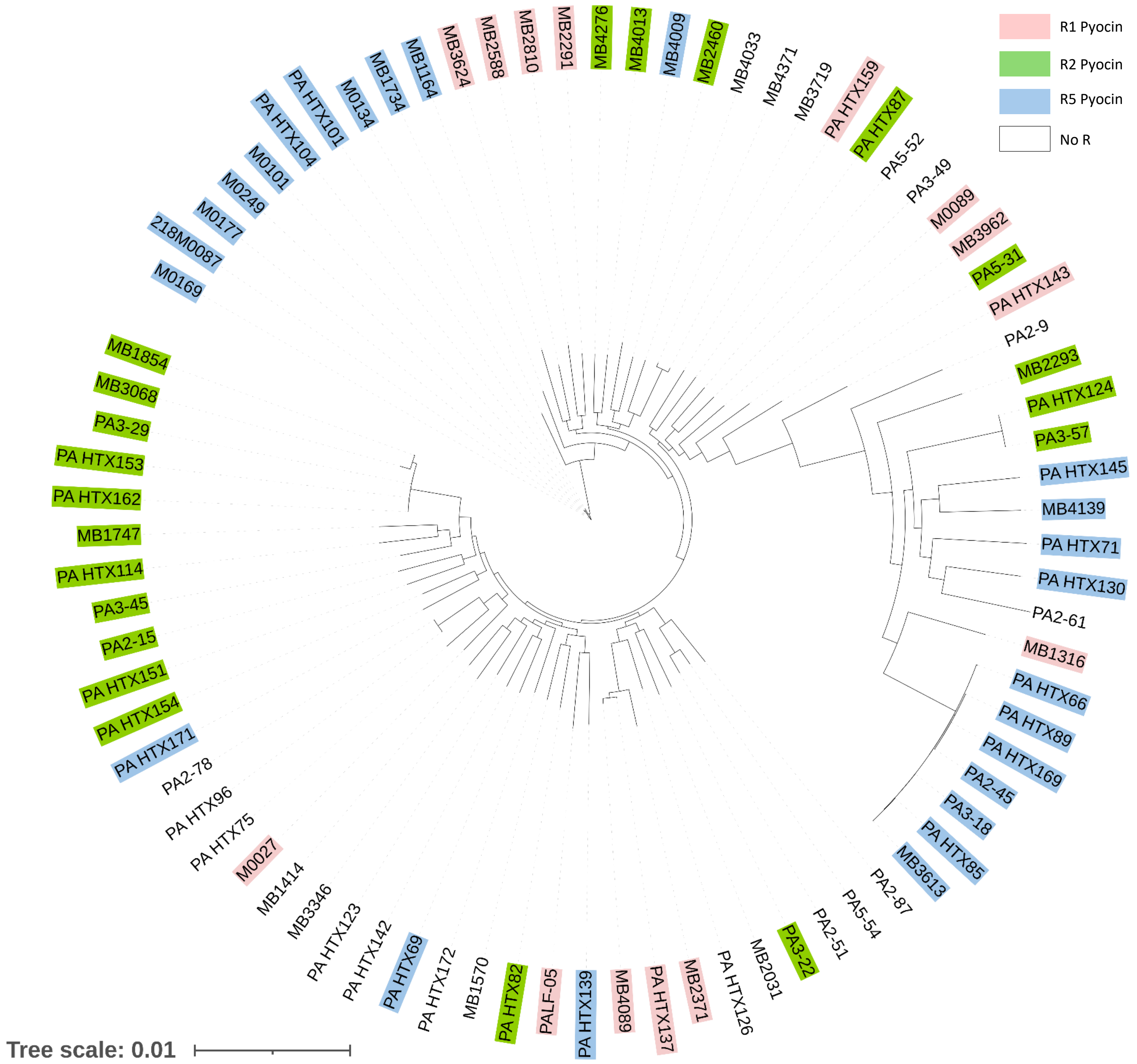

Figure S6. Branch length–scaled whole-genome phylogeny of *P. aeruginosa* isolates in phylogeny-based library. Circular maximum-likelihood tree generated from the core-genome SNP alignment (see Methods). Branch lengths are proportional to substitutions per site (scale bar = 0.01). Tip labels are highlighted in different colors by R pyocin subtype encoded by each isolate—R1 (pink), R2/3/4 (green), R5 (blue), and no R (white). This supplemental figure displays the true branch-length differences of the phylogeny for 81 clinical isolates in phylogeny-based library; the main-text figure is a topology-only cladogram (branch lengths not to scale) for readability.
